# Viral aetiology of bronchiolitis in hospitalised children in Qatar

**DOI:** 10.1186/s12879-017-2225-z

**Published:** 2017-02-13

**Authors:** Ibrahim Janahi, Anas Abdulkayoum, Fawziya Almeshwesh, Mohamed Alkuwari, Ahmed Al hammadi, Marwah Alameri

**Affiliations:** 1 Paediatric Pulmonology Unit, Hamad Medical Corporation, P. O. Box 3050, Doha, Qatar; 2 Icahn School of Medicine at Mount Sinai, New York, USA

**Keywords:** Length of stay, Bronchiolitis severity, Multivariate predictors

## Abstract

**Background:**

Bronchiolitis is considered one of the earliest and most common causes of hospitalisation in young children. Development of molecular technologies allowed a better understanding of bronchiolitis aetiology. Results from cohort studies evaluating the association between single, multiple viral infections and clinical outcomes are conflicting. Data on viral bronchiolitis in children were found to be limited in Qatar. This study aimed to determine frequency and seasonal trends of viral pathogens causing acute bronchiolitis, and to explore association between viral pathogens, disease severity and length of stay (LOS).

**Methods:**

This is a retrospective descriptive study, including children admitted in 2010 and 2011 with acute bronchiolitis. Presenting history, physical examination and respiratory viral co-infections as detected by molecular assays were analysed.

**Results:**

At least one virus was detected in 315/369 (85.4%) of included children with single and multiple viruses in 67 and 33% of cases respectively. Respiratory syncytial virus (RSV) was the most detected virus, accounting for 51.2% followed by rhinovirus (RV) in 25.5% of cases. Fall and summer admissions were associated with longer LOS. On multivariate logistic regression analysis, retraction (OR 3.96; 95% CI 1.64,9.59) and age group 1–3 months (OR 3.09; 95% CI 1.06,9.05) were associated with longer LOS. Crepitation (OR 9.15; 95% CI 1.58,53.13), retraction (OR 4.10; 95% CI 1.05,16.12) and respiratory rate (OR 1.46; 95% CI 1.28,1.66) were associated with moderate to severe bronchiolitis. Identifying the viral agent did not influence disease severity or LOS.

**Conclusion:**

Clinical presentation is of more relevance to LOS and disease severity than the detected viruses. Future studies should investigate the interplay between climate characteristics, population’s factors and the most detectable circulating viruses.

**Electronic supplementary material:**

The online version of this article (doi:10.1186/s12879-017-2225-z) contains supplementary material, which is available to authorized users.

## Background

Bronchiolitis is considered one of the earliest and most common causes of hospitalisation among young children during their first 2 years of life [1]. Although the causative agents include bacteria, fungi and viruses, this acute infection is mainly attributed to respiratory syncytial virus, accounting for 50–90% of the cases [2, 3]. With the current utilisation of reverse transcriptase real-time polymerase chain reaction (PCR), the detection of specific viral nucleic acids facilitated a better understanding of the viral aetiology of the infection. The 2014 American Academy of Paediatrics bronchiolitis guideline recommends against the routine use of radiographic or laboratory studies on the basis that knowing the infecting pathogen would rarely alter the clinical management [4]. However, a growing body of literature has identified an association between specific infecting pathogens with short and long-term outcomes [5]. Association between viral co-infection and disease severity have been assessed in several studies but with conflicting findings [5–7]. Although bronchiolitis is a self-limiting condition, hospitalisation rate has increased during the last two decades [3]. Assessment of bronchiolitis severity, through a combination of clinical symptoms and physical signs, remains a standard measure in daily practice though its impact on clinical outcomes, such as length of hospital stay, has yet to be confirmed. Data has been reported on seasonal variation of viral activity with conflicting evidence on its significance on disease severity and clinical outcomes [8, 9]. In Qatar, viral aetiology of bronchiolitis in children has been limited. Therefore the aims of this retrospective, descriptive study were: to determine the frequency and seasonal trends of viral pathogens causing acute bronchiolitis, and to explore the association between specific viral pathogens, disease severity and length of stay (LOS).

## Methods

### Study design

This retrospective descriptive study took place at the paediatric wards of Hamad General Hospital (HGH), a 603- bed, tertiary-care facility in Doha, for two consecutive years, 2010 and 2011. The institutional review board at Hamad Medical Corporation approved the study, IRB number 12054.

### Study population

The study population included children aged between 2 weeks- 2 years admitted to the paediatric ward at HGH with a diagnosis of acute bronchiolitis defined according to International Classification of Disease (ICD) code 466.1 in the 9^th^ revision of the ICD. Based on recorded clinical diagnosis, an episode of acute bronchiolitis was determined by a constellation of clinical signs and symptoms including fever, rhinitis, tachypnoea, cough, wheezing, crackles, use of accessory muscles and possible chest X ray findings of hyperinflation of the lungs, peri-bronchial thickening, collapsed segment or a lobe of the lung and increase interstitial markings. Due to the potential for an atypical natural history of bronchiolitis, children with the following diagnoses were excluded: born prematurely, defined as <37 weeks of gestation, those with low birth weight, defined as birth weight <2.5 kg, with underlying chronic lung disease or neurological disease or congenital heart disease, and those immunocompromised or with hospital-acquired bronchiolitis.

### Data collection

Medical charts of the children admitted to the paediatric ward during the study period were retrieved from the medical records department at HGH, a tertiary teaching hospital located in Doha, Qatar. A trained research staff screened patients’ discharge summaries for diagnosis of bronchiolitis. A data collection form (DCF) was developed to capture the necessary information. For every child who met the inclusion criteria the following data were collected: age, gender, family history of asthma and/or atopy, clinical presentation and vital signs upon admission and length of hospital stay. Bronchiolitis severity was based on a score ranging between 1 and 5 that assessed wheezing, retraction, respiratory rate and oxygen saturation (see Additional file 1: Table S1). Patients with score of 1 or 2 were considered to have mild bronchiolitis and to have moderate to severe bronchiolitis if score was between 3 and 4, with 5 indicating respiratory failure. Wheezing on physical exam was categorised as none, mild, moderate or severe. Retraction was categorised as none, intercostal, intercostal and subcostal or intercostal and subcostal with seesaw chest motion. Viral identification laboratory results, medical therapy and need of mechanical ventilation during hospital stay were recorded. Hospital admissions due to bronchiolitis were presented on an episode base.

### Nasopharyngeal sample collection and analysis

Nasopharyngeal samples were collected by inserting Dacron™ polyester tipped or FLOQSwab™ in both nostrils till the nasopharynx and rotating at 360°. The swabs were placed into 3 ml of transport medium (Copan, UTM-RT™, CA, USA). Following collection, samples were kept on wet ice and transported to central processing lab of HGH during the working hours. Specimens that were not delivered immediately to the lab were stored in a refrigerator (4 – 8 °C) for no more than 24 h. Beyond that, samples were stored at -70 °C and transported on dry ice. EZ1 virus mini kit v 2.0, was used for nucleic acid extraction (Qiagen®, Hilden, Germany). Multiplex, real-time, polymerase chain reaction (RT-PCR) using FTDResp21 kit (Fast Track Diagnostics, Silema, Malta) was used for the detection of respiratory pathogens on Applied Biosystem™ 7500 instrument (ThermoFisher Scientific Inc, MA, USA). The kit detects the following respiratory pathogens: influenza A and B; coronaviruses NL63, 229E, OC43 and HKU1; parainfluenza viruses 1, 2, 3 and 4; human metapneumovirus A/B (hMPV); rhinovirus (RV); respiratory syncytial virus A/B (RSV); adenovirus (AdV); enterovirus (EV); parechovirus (PeV); bocavirus (BoV) and *Mycoplasma pneumoniae* (Mpneu).

### Statistical analysis

The focus of the data analysis in our study was to determine frequency of each virus that is causing acute bronchiolitis in young children in our community and link it to the clinical presentation, length of hospital stay, severity of symptoms and need for intensive care support including invasive and non invasive ventilation. Categorical and continuous values were expressed as frequency (percentage) and mean ± SD or median and interquartile range (IQR) as appropriate. Descriptive statistics were used to summarise demographic, medical history, frequency of each virus and clinical characteristics of the patients. Associations between two or more qualitative variables were assessed using chi-square test and Fisher Exact test as appropriate. Quantitative data between the two independent groups were analysed using unpaired ‘t’ and Mann Whitney U tests as appropriate.

Univariate and multivariate logistic regression methods were used to assess and compute the predictive values of each predictor or risk factors for severity of bronchiolitis and hospital length of stay. For multivariate regression models, variables were considered if statistically significant at the *P* <0.10 level in univariate analysis or if determined to be clinically important. The results of logistic regression analyses were reported as odds ratio (OR) with 95% confidence intervals (CIs). Pictorial presentations of the key results were made using appropriate statistical graphs. A two-sided *P* value <0.05 was considered to be statistically significant. All statistical analyses were done using statistical packages SPSS 22.0 (SPSS Inc. Chicago, IL) and Epi Info 2000 (Centres for Disease Control and Prevention, Atlanta, GA).

## Results

### Patient identification and demographics

A total of 369 children were admitted to the hospital with a clinical diagnosis of bronchiolitis documented in their medical charts during the study period with 261 (75.2%) aged 3 months or younger and 247 (67%) were male sex as illustrated in Table 1. Of the 369 cases, 145 (39.3%) had a family history of atopy or asthma. On admission, 348 (94.3%) and 270 (73.2%) presented with cough and crepitation respectively, 261 (70.7%) had fever, while 251 (68.0%) and 231 (62.6%) experienced retracted breathing and wheezing respectively. Vitals signs (mean ± SD) including temperature 38.49 ± 0.80 °C, respiratory rate 62.51 ± 13.65 breaths per minute, pulse rate 167.88 ± 19.18 bpm and O_2_ saturation 96.40 ± 2.46% were recorded in the majority of the children. Generally, there were no differences in the clinical presentations between the males and the females. However, pertussis like symptoms were more frequently reported in females, 14/122 (11.5%), compared to males, 15/247 (6.1%).

**Table 1:** Demographic characteristics, clinical presentation and symptoms, viral etiology and management of children with acute bronchiolitis

Characteristics	Frequency (%)
Gender (*n* = 369)	
Male	247 (66.9%)
Female	122 (33.1%)
Age (months) (*n* = 347)	
< 1 months	144 (41.5%)
1 to 3 months	117 (33.7%)
> 3 months	86 (24.8%)
Clinical and presenting symptoms (*n* = 369)	
Cough	348 (94.3%)
Wheezing	231 (62.6%)
Rales	15 (4.1%)
Crepitation	270 (73.2%)
Retraction	251 (68.0%)
Fever	261 (70.7%)
Apnea	24 (6.5%)
Pertussis like symptoms	29 (7.9%)
Family history of atopy or asthma	145 (39.3%)
Max. Temperature (°C) (*n* = 353)	38.49 ± 0.80
Max. Respiratory rate (br/m) (*n* = 351)	62.51 ± 13.65
Max. Pulse (beat/m) (*n* = 351)	167.88 ± 19.18
O_2_ Saturation (%) (*n* = 341)	96.40 ± 2.46
Initial Clinical Severity Score (*n* = 349)	
1	19 (5.4%)
2	142 (40.7%)
3	159 (45.6%)
4	26 (7.4%)
5	3 (0.9%)
Nationality (*n* = 369)	
Qatari	234 (63.4%)
Non-Qatari	135 (36.6%)
Seasons (*n* = 355)	
Winter	154 (43.4%)
Spring	43 (12.1%)
Summer	20 (5.6%)
Fall	138 (38.9%)
Viral Etiology (*n* = 369)	
RSV	189 (51.2%)
Influenza A	3 (0.8%)
Influenza B	1 (0.3%)
ParaInfluenza 1	4 (1.1%)
ParaInfluenza 2	4 (1.1%)
ParaInfluenza 3	19 (5.1%)
ParaInfluenza 4	3 (0.8%)
Corona_NL63	3 (0.8%)
Corona_COC43	16 (4.3%)
Corona_229E	2 (0.5%)
Corona_HKU	2 (0.5%)
RV	94 (25.5%)
Enterovirus	6 (1.6%)
hMPV	23 (6.2%)
Boca Virus	15 (4.1%)
Parechovirus	11 (3.0%)
Adenovirus	23 (6.2%)
Other viruses	20 (5.4%)

*RSV* Respiratory syncytial virus, *RV* rhinovirus, *hMPV* Human metapneumovirus. Quantitative data were presented in mean ± SD. For some parameters the sum is not equal to total *n* = 369 due to some missing observations and respective % were computed based on non-missing observations

### Seasonal distribution

**Fig. 1:**
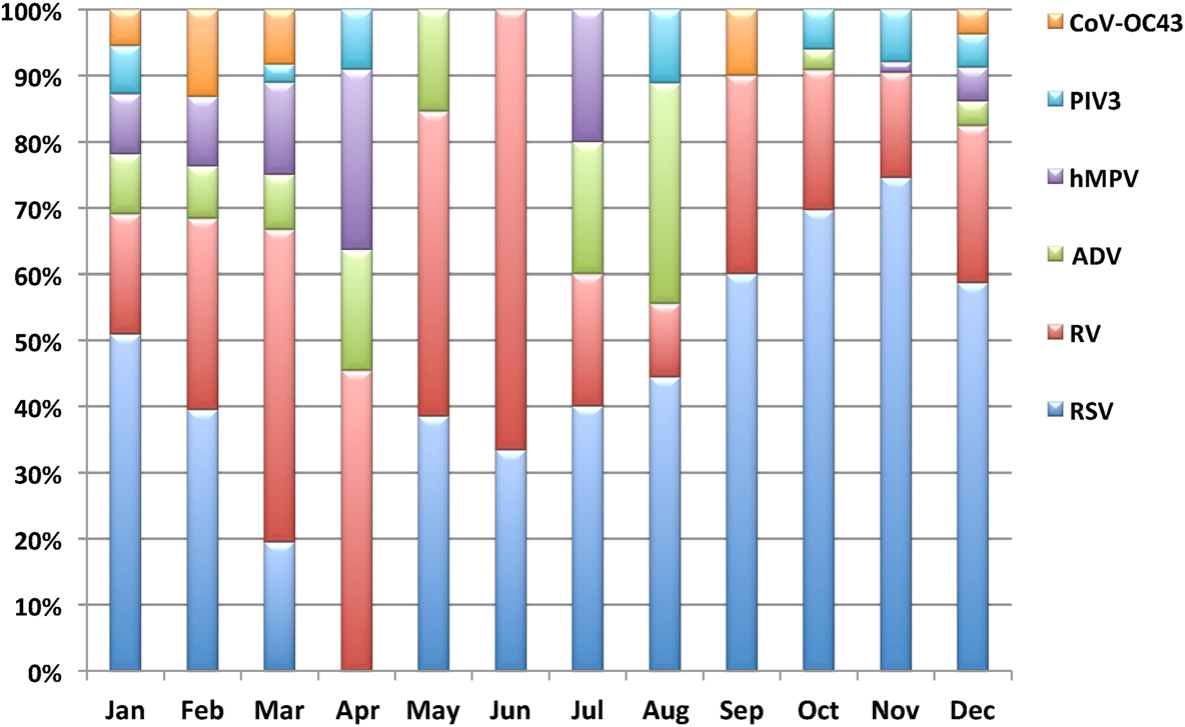
Monthly trends of respiratory viruses

The seasonal pattern of RSV and RV were generally distributed all year around although incidence of RSV infection peaked during the fall season whereas RV peaked during the spring. The findings of the current study revealed more adenovirus (ADV) infections in the summer months than spring (Fig. 1). Coronavirus (CoV-OC43) circulated predominantly during the winter months while parainfluenza virus 3 (PIV3) during the fall but sporadically across the year. Dual peaks were observed for human metapneumovirus (hMPV) during the spring and winter seasons.

**Fig. 2:**
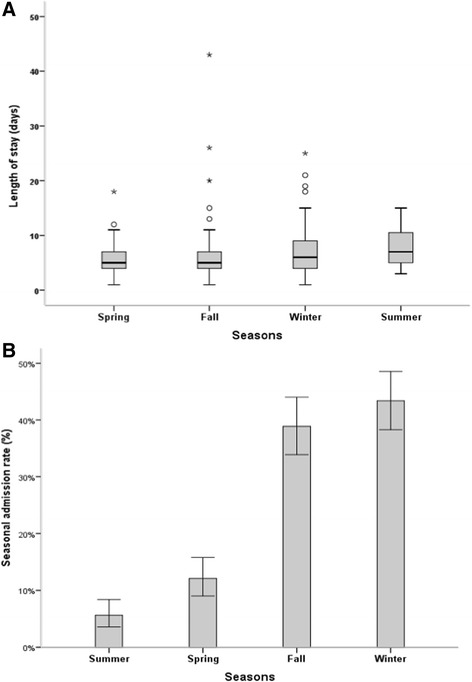
**a** Length of stay, in days, across different seasons. **b** Seasonal variations in hospital admission rates (%)

The median LOS was longer during the summer (7 days) compared to the winter (6 days) and shortest in the fall and spring (5 days), Fig. 2a. Our findings show a peak in hospital admission rates during the winter season, 154 (41.7%), followed by the fall, 138 (37.4%) with least during the summer season, 20 (5.4%) as presented in Fig. 2b.

### Viral aetiologies and multiple co-infections

We used polymerase chain reaction (PCR) to establish the viral aetiology of acute bronchiolitis. At least one virus was detected in 315/369 (85.4%) of children with single virus in 211/315 (67%) and multiple viruses in 104/315 (33.0%). RSV was the most common virus identified, accounting for 189 cases (51.2%) followed by RV in 94 cases (25.5%), 23 cases (6.2%) of hMPV and ADV each. Influenza A and B were the least detected viruses accounting for less than 1% of the cases, as shown in Table 1.

Out of the 315 samples analysed, 27.3% had dual infections and three viruses were detected in 5.4% of the sample with a quadruple infection found in one case. There were no marked differences in the management of single and multiple infections. β-agonists were used in the majority of cases, followed by antibiotics, hypertonic saline and adrenaline, presented in Table 2.

**Table 2:** Management and complications of acute bronchiolitis

Characteristics	Frequency (%)
Assisted ventilation	
Yes	32 (8.7%)
Assisted ventilation type (*n* = 32)	
Non-invasive	22 (6%)
Invasive	1 (0.3%)
Both	9 (2.4%)
Therapy/Treatment (*n* = 369)	
β agonist	331 (89.7%)
Adrenaline	151 (40.9%)
0.9% saline	42 (11.4%)
3.0% saline	160 (43.4%)
Steroids	85 (23%)
Antibiotics	239 (64.8%)
Mucomist	6 (1.6%)
Atrovent	38 (10.3%)
Complications (*n* = 369)	
Collapse	76 (20.6%)
Barotrauma	0 (0)
Atelectasis	97 (26.3%)
Pneumonia	63 (17.1%)
Sepsis	20 (5.4%)
Acute respiratory distress syndrome	0 (0)

### Respiratory viruses distribution by age-group, gender, medical history and clinical presentation

Children were grouped into three age groups with different rates of single and multiple viral infections, Table 3. In children aged <1 month, the most common detected virus was RSV (47.9%); for those between 1 and 3 months of age, RV (47.2%) was the most common virus. Children aged >3 months were found to be co-infected with RSV + RV (40%). Co-infection with RSV+ non-RV (75.8%) had the highest incidence in males whereas in females, RV+ non-RSV co-infection (40.9%) dominated. Family history of atopy or asthma, *P* = 0.008, and high respiratory rate, *P* = 0.026, were more common in RV single group, whereas crepitation was more frequent in the RSV+ non-RV co-infection group, *P* = 0.039. More children in the RV single group (91.7%) had LOS ≥4 days compared to the RSV single group (82.4%). Similar pattern was observed in terms of bronchiolitis severity with more children in the RV single group (65.7%) with moderate to severe bronchiolitis compared to children in the RSV single group (57.6%).

**Table 3:** Demographic characteristics, clinical presentations and seasonality of acute bronchiolitis by RSV, RV and other co-infections

		RSV (*n* = 127)	RV (*n* = 36)	RSV + RV (*n* = 25)	RSV+ any other non- RV (*n* = 33)	RV+ any other non-RSV (*n* = 22)	Others (*n* = 72)	*P*-value*
Gender	Male	76 (59.8)	27 (75)	15 (60)	25 (75.8)	13 (59.1)	53 (73.6)	0.180
	Female	51 (40.2)	9 (25)	10 (40)	8 (24.2)	9 (40.9)	19 (26.4)	
Age Group	<1 months	57 (47.9)	14 (38.9)	4 (16)	8 (25)	8 (36.4)	28 (42.4)	0.064
	1 to 3 months	33 (27.7)	17 (47.2)	11 (44)	12 (37.5)	8 (36.4)	23 (34.8)	
	>3 months	29 (24.4)	5 (13.9)	10 (40)	12 (37.5)	6 (27.3)	15 (22.7)	
Nationality	Qatari	90 (70.9)	22 (61.1)	16 (64)	22 (66.7)	11 (50)	42 (58.3)	0.335
Clinical and presenting symptoms	Cough	124 (100)	35 (97.2)	25 (100)	32 (97)	22 (100)	66 (97.1)	0.408
	Wheezing	83 (68)	26 (72.2)	20 (83.3)	22 (68.8)	13 (59.1)	42 (62.7)	0.471
	Crepitation	88 (78.6)	30 (85.7)	22 (91.7)	25 (96.2)	16 (76.2)	56 (93.3)	*0.039*
	Retraction	89 (76.7)	29 (82.9)	18 (75)	28 (90.3)	13 (59.1)	50 (82)	0.121
	Fever	90 (74.4)	25 (71.4)	19 (79.2)	28 (87.5)	16 (80)	51 (79.7)	0.621
	Apnea	8 (7.3)	1 (3.1)	3 (13)	1 (4.2)	1 (5.3)	8 (13.8)	0.415
	Pertussis like symptoms	9 (8.3)	3 (11.5)	5 (20)	2 (8.3)	3 (15.8)	5 (10.2)	0.613
	Family history of atopy or asthma	54 (61.4)	20 (69)	10 (45.5)	14 (56)	3 (16.7)	29 (59.2)	*0.008*
	Max. temperature (degree C)	38.49 ± 0.82	38.52 ± 0.88	38.64 ± 0.77	38.49 ± 0.79	38.63 ± 0.74	38.36 ± 0.84	0.687
	Max. respiratory rate (Br/min)	61.3 ± 9.5	67.5 ± 20.1	60.3 ± 9.4	62.3 ± 7.1	59.6 ± 8.0	66.8 ± 20.5	*0.026*
	Max. Pulse (Beat/min)	167.5 ± 15.2	170 ± 34	172.2 ± 14.8	164.2 ± 16.2	169 ± 10.8	168.4 ± 23.1	0.750
	O2 Saturation	96.57 ± 2.40	95.25 ± 2.73	96.55 ± 1.60	95.8 ± 3.48	96.29 ± 1.87	96.42 ± 2.24	0.087
ICS score	3 to 4	72 (57.6)	23 (65.7)	14 (58.3)	17 (56.7)	10 (45.5)	34 (51.5)	0.692
Seasons	Winter	44 (34.9)	17 (47.2)	14 (56)	13 (39.4)	11 (50)	27 (40.9)	0.483
	Spring	14 (11.1)	6 (16.7)	4 (16)	3 (9.1)	0 (0)	9 (13.6)	
	Summer	12 (9.5)	1 (2.8)	1 (4)	2 (6.1)	2 (9.1)	2 (3)	
	Fall	56 (44.4)	12 (33.3)	6 (24)	15 (45.5)	9 (40.9)	28 (42.4)	
Assisted ventilation	Yes	12 (10)	6 (16.7)	1 (4.3)	2 (6.1)	2 (9.5)	8 (12.3)	0.642
LOS (days)	≥4 days	103 (82.4)	33 (91.7)	21 (87.5)	30 (90.9)	19 (86.4)	56 (84.8)	0.703

*LOS* Length of stay in hospital, *ICS* Initial clinical severity score. *P-values computed using one-way analysis of variance (ANOVA). Chi-square and Yates corrected Chi-square statistical tests methods

### Analysis of risk factors of length of hospital stay and bronchiolitis severity

A total of 291 (82.4) had a LOS ≥4 days. On univariate logistic regression analysis, admission during the fall season (unadjusted OR 1.89; 95% CI 1.01,3.53; *P* = 0.046), cough (unadjusted OR 7.27; 95% CI 1.2,44.5; *P* = 0.032), retraction (unadjusted OR 2.70; 95% CI 1.5,5.1; *P* = 0.002), pulse rate (unadjusted OR 1.01; 95% CI 1.0,1.03; *P* = 0.011) were significantly associated with longer LOS as shown in Table 4. Oxygen saturation level (unadjusted OR 0.78; 95% CI 0.66,0.89; *P* = 0.001) was associated with shorter LOS. Detection of RV, RSV+ non-RV, age group 1–3 months and admission during the summer season increased LOS but these differences did not reach statistical significance (*P* >0.05). On multivariate logistic regression analysis, the only factors that remained statistically significant were retraction (adjusted OR 3.96; 95% CI 1.64,9.59; *P* = 0.002), age group 1–3 months (adjusted OR 3.09; 95% CI 1.06,9.05; *P* = 0.039) and high respiratory rate (adjusted OR 1.07; 95% CI 1.0,1.14; *P* = 0.05), provided in Additional file 1: Table S2.

**Table 4:** Predictors of length-of-stay ≥4 days among children with mild to severe bronchiolitis: logistic regression analysis

	LOS <4 days	LOS ≥ 4 days	Odds Ratio (OR) (95% CI)	*P*-Value
Demographic characteristics				
Age group				
≤ 1 month	25 (42.4%)	118 (41.5%)	1.36 (0.70, 2.7)	0.369
1 to 3 months	15 (25.4%)	100 (35.2%)	1.92 (0.91, 4.0)	0.086
> 3 months	19 (32.2%)	66 (23.2%)	1.0 (Reference)	
Male	39 (62.9%)	196 (67.4%)	1.22 (0.69, 2.15)	0.500
Female	23 (37.1%)	95 (32.6%)	1.0 (Reference)	
Qatari	38 (61.3%)	185 (63.6%)	1.1 (0.63, 1.9)	0.735
Non-Qatari	24 (38.7%)	106 (36.4%)	1.0 (Reference)	
Types of viruses, seasonal trend and severity				
RSV	22 (50%)	103 (39.3%)	1.0 (Reference)	
RV	3 (6.8%)	33 (12.6%)	2.35 (0.66, 8.35)	0.187
RSV + RV	3 (6.8%)	21 (8%)	1.50 (0.41, 5.46)	0.542
RSV+ any other non-RV	3 (6.8%)	30 (11.5%)	2.14 (0.60, 7.63)	0.243
RV+ any other non-RSV	3 (6.8%)	19 (7.3%)	1.35 (0.37, 4.97)	0.649
Others	10 (22.7%)	56 (21.4%)	1.20 (0.53, 2.70)	0.667
Seasons				
Spring	8 (12.9%)	35 (12%)	1.25 (0.53, 2.90)	0.610
Summer	2 (3.2%)	18 (6.2%)	2.57 (0.57, 11.6)	0.220
Fall	18 (29%)	119 (41%)	1.89 (1.01, 3.53)	*0.046*
Winter	34 (54.8)	119 (40.9)	1.0 (Reference)	
ICS score: 3 to 4	27 (43.5%)	156 (55.5%)	1.62 (0.93, 2.82)	0.089
ICS score: 1 to 2	35 (56.5%)	125 (44.5%)	1.0 (Reference)	
Medical history and physical exam findings				
Cough	59 (95.2%)	286 (99.3%)	7.27 (1.2, 44.5)	*0.032*
Wheezing	40 (66.7%)	189 (66.5%)	0.99 (0.55, 1.8)	0.986
Crepitation	42 (76.4%)	226 (85.6%)	1.84 (0.90, 3.7)	0.092
Retraction	33 (60%)	215 (80.2%)	2.70 (1.5, 5.1)	*0.002*
Fever	47 (78.3%)	212 (76.3%)	0.89 (0.45, 1.74)	0.731
Apnea	3 (5.6%)	21 (8.4%)	1.56 (0.45, 5.4)	0.485
Pertussis like symptoms	3 (5.5%)	26 (11%)	2.15 (0.63, 7.4)	0.225
Family history of asthma	22 (55%)	121 (56.3%)	1.1 (0.53, 2.1)	0.881
Maximum temperature (°C)	38.39 ± 0.87	38.52 ± 0.78	1.22 (0.86, 1.73)	0.255
Maximum respiratory rate (br/m)	60.34 ± 15.13	62.99 ± 13.32	1.02 (0.99, 1.1)	0.160
Maximum pulse rate (beat/m)	161.8 ± 22.1	169.1 ± 18.1	1.01 (1.0, 1.03)	*0.011*
O_2_ saturation (%)	97.37 ± 1.84	96.18 ± 2.53	0.78 (0.66, 0.89)	*0.001*

*LOS* Length of stay in hospital, *ICS* Initial clinical severity score, *OR* odds ratio, *CI* confidence interval. Dichotomous outcome LOS <4 days considered as reference group

A total of 185 (53.4%) of the hospitalised children experienced moderate to severe bronchiolitis. On univariate logistic regression analysis, children in this group required greater need for respiratory support (OR 5.85; 95% CI 1.98,17.3; *P* = 0.001), experienced more wheezing (OR 1.82; 95% CI 1.15,2.89; *P* = 0.010), crepitation (OR 3.11; 95% CI 1.64,5.90; *P* = 0.001), and retraction (OR 5.65; 95% CI 3.13,10.2; *P* <0.001), increased respiratory rate (OR 1.09; 95% CI 1.07,1.12; *P* <0.001), increased pulse rate (OR 1.03; 95% CI 1.02,1.04; *P* < 0.001), but higher oxygen saturation levels reduced the odds of moderate to severe bronchiolitis (OR 0.85; 95% CI 0.77,0.93; *P* = 0.001) as presented in Table 5. On multivariate logistic regression analysis, crepitation (adjusted OR 9.15; 95% CI 1.58,53.13; *P* = 0.014), retraction (OR 4.10; 95% CI 1.05,16.12; *P* = 0.043), and increased respiratory rate (adjusted OR 1.46; 95% CI 1.28,1.66; *P* < 0.001) were significant predictors associated with bronchiolitis severity, provided in Additional file 1: Table S3. Complications including atelectasis, collapse, pneumonia and sepsis were reported in 97 (26.3%), 76(20.6%), 63(17.1%) and 20(5.4%) of hospitalised children respectively.

**Table 5:** Predictors of mild to severe ICS score among children with viral bronchiolitis: logistic regression analysis

	ICS score: 1 to 2 (mild)	ICS score: 3 to 4 (moderate to severe)	Odds Ratio (OR) (95% CI)	*P*-Value
Demographic characteristics				
Age group				
≤ 1 month	70 (44.0%)	68 (38.4%)	0.84 (0.49, 1.45)	0.535
1 to 3 months	50 (31.4%)	64 (36.2%)	1.11 (0.63, 1.95)	0.720
> 3 months	39 (24.5%)	45 (25.4%)	1.0 (Reference)	
Male	101 (62.7%)	131 (70.8%)	1.44 (0.92, 2.26)	0.111
Female	60 (37.3%)	54 (29.2%)	1.0 (Reference)	
Qatari	97 (60.2%)	123 (66.5%)	1.31 (0.84, 2.03)	0.229
Non-Qatari	64 (39.8%)	62 (33.5%)	1.0 (Reference)	
Types of viruses, seasonal trend and severity				
RSV	53 (40.2%)	72 (42.4%)	1.0 (Reference)	
RV	12 (9.1%)	23 (13.5%)	1.41 (0.65, 3.09)	0.389
RSV + RV	10 (7.6%)	14 (8.2%)	1.03 (0.42, 2.50)	0.947
RSV+ any other non-RV	13 (9.8%)	17 (10%)	0.96 (0.43, 2.15)	0.926
RV+ any other non-RSV	12 (9.1%)	10 (5.9%)	0.61 (0.25, 1.53)	0.293
Others	32 (24.2%)	34 (20%)	0.78 (0.43, 1.42)	0.421
Seasons				
Winter	62 (38.5%)	88 (47.8%)	1.0 (Reference)	
Spring	23 (14.3%)	19 (10.3%)	0.58 (0.29, 1.16)	0.124
Summer	6 (3.7%)	13 (7.1%)	1.53 (0.55, 4.24)	0.417
Fall	70 (43.5%)	64 (34.8%)	0.64 (0.40, 1.03)	0.066
LOS ≥ 4 days^a^	125 (78.1%)	156 (85.2%)	1.62 (0.93, 2.82)	0.089
Assisted Ventilation^a^	4 (2.6%)	24 (13.4%)	5.85 (1.98, 17.3)	*0.001*
Medical history and physical exam findings				
Cough	158 (98.8%)	181 (98.4%)	0.76 (0.13, 4.63)	0.769
Wheezing	95 (60.1%)	132 (73.3%)	1.82 (1.15, 2.89)	*0.010*
Crepitation	109 (75.7%)	155 (90.6%)	3.11 (1.64, 5.90)	*0.001*
Retraction	87 (60.8%)	158 (89.8%)	5.65 (3.13, 10.2)	*<0.001*
Fever	118 (75.6%)	135 (76.3%)	1.03 (0.63, 1.71)	0.893
Apnea	13 (9.3%)	9 (5.6%)	0.58 (0.24, 1.40)	0.224
Pertussis like symptoms	12 (9.2%)	16 (10.1%)	1.12 (0.51, 2.46)	0.782
Family history of asthma	59 (52.7%)	81 (58.3%)	1.26 (0.76, 2.07)	0.375
Maximum temperature (°C)	38.44 ± 0.82	38.56 ± 0.79	1.20 (0.92, 1.56)	0.156
Maximum respiratory rate (br/m)	57.98 ± 17.17	66.37 ± 7.83	1.09 (1.07, 1.12)	*<0.001*
Maximum pulse rate (beat/m)	163.2 ± 19.9	171.9 ± 17.7	1.03 (1.02, 1.04)	*<0.001*
O_2_ saturation (%)	96.91 ± 2.01	96.02 ± 2.64	0.85 (0.77, 0.93)	*0.001*

*LOS* Length of stay in hospital, *OR* odds ratio, *CI* confidence interval, *ICS* Initial clinical severity score. ^a^Reference category: LOS <4 days; not assisted ventilation. Dichotomous outcome ICS score value 1 to 2 was taken as reference group

## Discussion

To our knowledge this is the first comprehensive report on viral aetiology in hospitalised children with bronchiolitis in the State of Qatar. Our study revealed that RSV and RV were the most commonly detected causative agents. This finding is in contrast to a previous report with a small sample size of 56 children with respiratory tract infection (RTI) in Qatar in which hMPV was the most frequently isolated virus [10]. Our findings are, however, in line with a number of large studies conducted in the Middle East and elsewhere [11–14]. In Jordanian children of less than 2 years of age, RSV was a major cause of hospitalisation [11]. Other studies conducted in Saudi Arabia and Turkey reported the same [15, 16].

Geographic variations in seasonal activity of the most commonly detected respiratory viruses have been reported in the literature. In temperate countries, RSV peaks during the winter season, but with variation in the tropics. This diversity is mainly attributed to the region’s climate characteristics, i.e. relative humidity, average monthly temperature and rainfall [17–19]. In their study, Haynes et al. assessed RSV seasonality in seven countries with diverse climate characteristics and found that RSV onset and peak timings were inconsistent with climate characteristics in all assessed countries apart from Thailand. RSV offset was found to be consistent in Guatemala [20]. Rhinovirus A was detected all year around with high proportion by the onset of summer in Japan but during the fall and spring in Cyprus while Rhinovirus C was mainly detected in the winter but during the fall and spring in Japan and Cyprus, respectively [18, 21].

In this study, RSV exhibited a strong seasonal pattern with peak activity during the fall and summer months, in contrast to RV, which showed minimal circulation during the fall.

In comparison to children with RSV only infections, children infected with RSV in combination with non-RV or RV alone were more likely to have longer LOS. However, we found no significant association between single or multiple infections and disease severity or LOS. The findings are consistent with two large studies conducted in North America and Europe [13, 14], but in contrast with previous reports where RV was associated with shorter LOS [12, 22]. These discrepancies might be related to sample size, study design and variation in host risk factors.

In this study, we noted an association between individual components of the clinical severity score and bronchiolitis outcome after adjusting for clinical and demographic factors. Similar to Ricart et al., McCallum et al. and Weisgerber et al., the data indicate that, in the Qatari community, children’s clinical characteristics are more relevant than the specific infecting viruses in determining the duration of LOS and disease severity [23–25].

Detection of the presence of multiple infections has become common in practice although its clinical impact on disease management and predicting outcomes remains unclear [26]. By using RT-PCR to quantify viral load, a number of studies have concluded that RSV genomic load is correlated with disease severity [13, 14]. Similarly, two investigational anti-RSV drugs showed promising results in reducing viral load and clinical disease severity [27, 28].

Some studies have determined severity based on a modified Wood-Downes score or the need for paediatric intensive care unit (PICU) admission [7, 23]. In this study we analysed severity based on a clinical score that allows children’s classification according to respiratory rate, oxygen saturation and physical examination, mirroring the severity of the respiratory burden.

Previous studies have reported males to be more susceptible than females to lower RTIs across different age groups [29]. In this study the percent of males with severe bronchiolitis was as twice as that to females, 70.8 vs. 29.2%. An explanation was suggested two decades ago by Gupta et al. in which the disproportionally narrower peripheral airways in the younger males could be a possible mechanism of the observed difference [30].

Childhood respiratory infection may influence the development of chronic respiratory diseases. In a cohort of 47 Swedish hospitalised infants with RSV-bronchiolitis, asthma was prevalent in 30% of the group when aged 7 years increasing to 37% at the age of 13 [31]. While Jackson et al. noted an increase in asthma risk in children with RSV associated wheezing; RV was associated with a 10-fold increase of asthma [32]. An earlier retrospective study from Qatar noted an increase in recurrent wheezing by 44% in hospitalised infants due to RSV bronchiolitis 2 years after admission compared to 12% in the control group [33]. In the current study, 63% of the admitted children had wheezing of which 51.2% had RSV associated bronchiolitis. Compared to RSV, RV associated bronchiolitis is suggested to occur in older infants, 13 versus 5 months, who present more often with atopic dermatitis and blood eosinophilia [34].

This study has potential limitations. First, it is a retrospective study at a single hospital. Another limitation is that data on concomitant bacterial infection, viral genomic load and palivizumab prophylaxis were not collected.

## Conclusions

In summary, RSV was the most common virus identified, followed by RV with peaks during the fall and spring seasons respectively. Our data show that the clinical presentation is more related to the duration of hospital stay and disease severity than the detected viruses.

Conducting a prospective, multi site surveillance of viral bronchiolitis in the warm, desert climate of the Gulf Cooperation Council (GCC) countries will allow for proper timing of preventable measures. Future studies within the GCC countries should investigate the interplay between climate characteristics, population’s factors and the most detectable circulating viruses.

## Additional file

Additional file 1: Table S1. Bronchiolitis severity score; **Table S2.** Predictors of hospital length-of-stay ≥ 4 days among children with mild to severe bronchiolitis associated with different viruses: multivariate logistic regression analysis; **Table S3.** Predictors of moderate (1 to 2) to severe (3 to 4) ICS score among children with viral aetiology of acute bronchiolitis: multivariate logistic regression analysis. (DOCX 33 kb)

## Abbreviations

ADV: Adenovirus
CI: Confidence interval
CoV-OC43: Coronavirus
DCF: Data collection form
GCC: Gulf cooperation council
HGH: Hamad general hospital
hMPV: Human metapneumovirus
ICD: International classification of disease
IQR: Interquartile range
IRB: Institutional review board
LOS: Length of stay
OR: Odds ratio
PCR: Polymerase chain reaction
PICU: Paediatric intensive care unit
PIV3: Parainfluenza virus 3
RSV: Respiratory syncytial virus
RTI: Respiratory tract infection
RV: Rhinovirus
