# Supplementary material for: Viral aetiology of bronchiolitis in hospitalised children in Qatar
Source: BMC Infect Dis. 2017 Feb 13;17:139. doi: 10.1186/s12879-017-2225-z (PMC5307797; doi:10.1186/s12879-017-2225-z)
Supplement: Additional file 1: Table S1. — Bronchiolitis severity score; Table S2. Predictors of hospital length-of-stay ≥ 4 days among children with mild to severe bronchiolitis associated with different viruses: multivariate logistic regression analysis; Table S3. Predictors of moderate (1 to 2) to severe (3 to 4) ICS score among children with viral aetiology of acute bronchiolitis: multivariate logistic regression analysis. (DOCX 33 kb) [file 12879_2017_2225_MOESM1_ESM.docx]

**Table S1.** Bronchiolitis severity score

| **Score 0** | Well or baseline clinical condition |
| --- | --- |
| **Score 1** | Respiratory rate/min < 45; oxygen saturation = 95 percent; adventitial (wheezing, rales)-none; and retractions-none |
| **Score 2** | Respiratory rate/min of 45 to 59; oxygen saturation 91 to 94 percent; adventitial (wheezing, rales)-mild; and retractions-intercostal |
| **Score 3** | Respiratory rate/min of 60 to 74; oxygen saturation of 86 to 90 percent; adventitial (wheezing, rales)-moderate; and retractions-intercostal and subcostal |
| **Score 4** | Respiratory rate/min of 75+; oxygen saturation = 85 percent; adventitial (wheezing, rales)-severe; and retractions-intercostal and subcostal with seesaw chest motion |
| **Score 5** | Respiratory failure |

**Table S2.** Predictors of hospital length-of-stay ≥4 days among children with mild to severe bronchiolitis associated with different viruses: Multivariate logistic regression analysis

LOS: Length of stay in hospital; OR: odds ratio; CI: confidence interval; LOS <4 days considered as reference group.

|  | **LOS <4 days** | **LOS ≥ 4 days** | **Adjusted odds ratio (OR) (95%CI)** | **P-Value** |
| --- | --- | --- | --- | --- |
| Age group  ≤ 1 month  1 to 3 months  >3 months | 25 (42.4%)  15 (25.4%)  19 (32.2%) | 118 (41.5%)  100 (35.2%)  66 (23.2%) | 2.14 (0.86, 5.33)  3.09 (1.06, 9.05)  1.0 (Reference) | 0.103  **0.039** |
| Retraction | 47 (60%) | 215 (80.2%) | 3.96 (1.64, 9.59) | **0.002** |
| Maximum respiratory rate | 60.34±15.13 | 62.99±13.32 | 1.07 (1.0, 1.14) | 0.05 |

**Table S3.** Predictors of moderate (1 to 2) to severe (3 to 4) ICS score among children with viral etiology of acute bronchiolitis: multivariate logistic regression analysis

LOS: Length of stay in hospital; OR: odds ratio; CI: confidence interval. ICS: Initial clinical severity. Dichotomous outcome variable; ICS score value 1 to 2 was taken as reference group.

|  | **ICS score:**  **1 to 2 (mild)** | **ICS score: 3 to 4**  **(moderate to severe)** | **Adjusted odds ratio (OR) (95%CI)** | **P-Value** |
| --- | --- | --- | --- | --- |
| Crepitation | 109 (75.7%) | 155 (90.6%) | 9.15 (1.58, 53.13) | **0.014** |
| Retraction | 87 (60.8%) | 158 (89.8%) | 4.10 (1.05, 16.12) | **0.043** |
| Family history of asthma | 59 (52.7%) | 81 (58.3%) | 2.61 (0.78, 8.80) | 0.121 |
| Maximum respiratory rate | 57.98±17.17 | 66.37±7.83 | 1.46 (1.28, 1.66) | **<0.001** |
